# Awareness of periodontitis and its relationship with systemic health among undergraduate medical students at a teaching hospital in Nepal: A cross-sectional study

**DOI:** 10.1371/journal.pone.0321315

**Published:** 2025-04-16

**Authors:** Bibek Kattel, Abhishek Kumar, Akash Kumar Giri, Pujan Acharya, Santosh Kumari Agrawal

**Affiliations:** 1 Department of Periodontology & Oral Implantology, College of Dental Surgery, BP Koirala Institute of Health Sciences, Dharan, Nepal; 2 Department of Public Health Dentistry, College of Dental Surgery, BP Koirala Institute of Health Sciences, Dharan, Nepal; King Faisal University, Saudi Arabia

## Abstract

**Background:**

Periodontitis is a multifactorial chronic inflammatory disease primarily caused by bacterial plaque, but may be affected by the host immune response, diabetes, inadequate nutrition, smoking and stress.

The systemic implications of periodontitis, particularly its associations with cardiovascular diseases, diabetes mellitus, and respiratory infections, highlight the importance of medical students acquiring in-depth insight into this condition. This knowledge is essential for promoting both oral and general health in the public.

**Objective:**

To assess the awareness of periodontitis and its relationship with systemic health among undergraduate medical students at a teaching hospital in Nepal.

**Materials and methods:**

A structured self-administered questionnaire was used to assess awareness of periodontitis and its relationship with systemic health. A cross-sectional study was conducted among 3rd-, 4th-, and final-year medical students and interns at the BP Koirala Institute of Health Sciences, Dharan, from January 2024 to March 2024. The data were analyzed via descriptive statistics in SPSS version 27.

**Results:**

A total of 218 participants were included in the study with a mean age of 24.03 ± 1.85 years. The majority (76.1%) of the students were male. Approximately 95.9% (209) of the participants had heard of term periodontitis. The majority of participants identified bad breath (83.5%), gum recession (78.4%), bleeding gums (85.3%), and loose teeth (71.1%) as symptoms of periodontitis, with more than 80% correctly recognizing gum recession in all groups except for 3rd-year students (52.8%). The majority of the students were aware of the risk factors associated with periodontitis.

**Conclusion:**

There was a notable gap in the recognition of symptoms, risk factors, and systemic links related to periodontitis among 3rd year students compared with 4th year, final year students and interns. This highlights the need for improved education on the topic among 3rd and 4th year medical students.

## Introduction

Periodontal disease, which includes gingivitis and periodontitis, is considered one of the most prevalent diseases in the population, worldwide. If not handled, it may lead to tooth loss [1,2]. Periodontal disease is caused primarily by bacterial plaque; however, host immune response, diabetes, inadequate nutrition, smoking, and stress can affect the genesis and progression of both gingival and periodontal disorders [3].

Dental plaque, which is now considered a biofilm, is the prime cause of inflammation in and around tooth-supporting structures [4]. Periodontal disease begins and progresses due to an imbalance in the normal oral microbiota found in dental plaque. This imbalance interacts with the host’s immune defenses, causing inflammation and disease. This condition continues in cycles of activity and inactivity until either the affected tooth is extracted or the microbial biofilm is therapeutically removed, thus reducing inflammation [5].

In Nepal, 52.5% of the population is affected by gingivitis, while 47.5% suffer from periodontitis, indicating a higher prevalence in developing countries than in developed countries [6]. According to a recent assessment, the prevalence of gingivitis varies between 5% to 100% worldwide [7]. This discrepancy emphasizes how localized research is necessary to comprehend Nepal’s particular risk factors and difficulties. Our study intends to close this gap by offering vital population-specific data that will guide focused public health initiatives and efficient methods for managing periodontal disease. The first clinical symptom suggestive of gingivitis, which most patients present with is bleeding while brushing. However, more severe forms of periodontitis can lead to loss of alveolar bone and ultimately, loosening of teeth [8].

Recent studies have explored the connections between periodontal health and other systemic conditions such as obesity, anxiety, stress, and kidney disease. According to the World Health Organization (WHO), oral diseases, including periodontal diseases (PDs), are a significant component of overall health [9]. Williams and Offenbacher emphasized that periodontal disease is influenced by systemic conditions such as diabetes, cardiovascular and respiratory diseases, and adverse pregnancy outcomes. They introduced “periodontal medicine” to highlight the impact of these systemic diseases on periodontal health, beyond the role of dental plaque alone [10].

Periodontal infections are recognized not only for their impact on oral health but also for their systemic implications. The chronic inflammation and bacterial burden associated with periodontal infections can lead to the release of inflammatory mediators into the bloodstream, potentially exacerbating systemic conditions such as cardiovascular disease and diabetes mellitus. Conversely, systemic health conditions like diabetes and immunosuppression can compromise the body’s ability to combat oral infections, thereby increasing the risk and severity of periodontal disease [4].

Medical students are most likely to encounter vulnerable populations and should be aware of this information to influence the course of systemic diseases and determine appropriate treatments and prognoses for a holistic approach to patient care [11]. This interplay underscores the necessity of integrated oral and systemic healthcare approaches to mitigate both the oral and systemic consequences of periodontal infections [12].

## Methods

### Study design and ethical considerations

A descriptive cross-sectional study was conducted among 218 medical students at BP Koirala Institute of Health Sciences, Dharan, from January 2024 to March 2024. Ethical approval was obtained from the Departmental Research Unit (DRU/86/023). Written informed consent was obtained from all participants.

### Study population

The participants included 3rd-year, 4th-year, final-year medical students, and interns. Inclusion criteria required students to be enrolled in the MBBS program and willing to participate.

### Data collection tool

A structured, self-administered questionnaire comprising 11 questions was adopted from a study conducted by Adam et al. [12]. Three questions addressed demographics, while nine focused on awareness of periodontitis and its systemic links. The questionnaire was developed on the basis of previous studies and validated through a pilot study with 20 participants, ensuring reliability and clarity.

### Sample size calculation

The sample size was calculated using the following formula:


$$=\frac{z_{1-\alpha /2}^{2}pq}{l^{2}}$$

Here, z_1−α/2_=value at a specific confidence level (It is 1.96 when α is 0.5% at 95% confidence level).

p = proportion of the event in the population [Here, p = 0.84; taken from a study done by Adam et al. [12] The estimated awareness level of periodontitis among medical students is represented by this value, which shows that 84% of research participants were aware of the term and its meaning.

q = p-1

*l*=acceptable margin of error in estimating the true population proportion.


$$=\frac{z_{1-\alpha /2}^{2}pq}{l^{2}}$$

$$=\frac{4\times 0.84\times \left(1-0.84\right)}{0.05^{2}}=215.04\approx 215$$

### Statistical analysis

After their entry into Microsoft Excel 2019, the data were analyzed using SPSS version 27. Descriptive statistics (frequencies, percentages, means) were used to summarize the data. Chi-square tests and ANOVA were used to compared groups, with significance set at p < 0.05.

## Results

### Participant demographics

**Fig 1:**
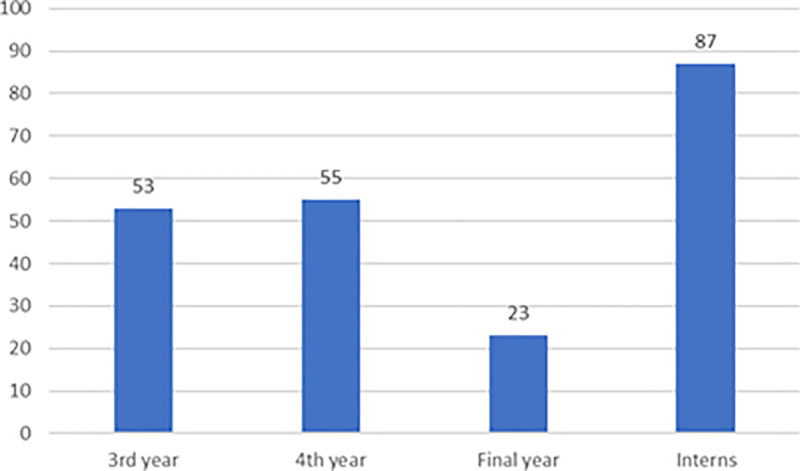
Number of participants from 3^rd^, 4^th^, final year and interns.

A total of 218 (N) participants [53 from the 3rd year, 55 from the 4th year, 23 from the final year, and 87 (interns) responded to the questionnaire [Fig 1 and Table 1]. The majority of the participants were male (166; 76.1%). The mean age of the study participants was 24.03 ± 1.85 years, ranging from 20 to 30 years.

**Table 1:** Total number of participants (N=218).

Respondent Group	Number of Respondents (N)
**3rd Year**	53
**4th Year**	55
**Final Year**	23
**Interns**	87
**Total**	218

### Awareness of periodontitis

Among the total participants, 209 (95.9%) had already heard of the term ‘periodontitis’. The highest percentage, 6 (11.3%) out of 53 students, in the 3rd year had never heard of the term ‘periodontitis’ [Table 2].

**Table 2:** Awareness of periodontitis (N=218).

Question	Response	3rd Year	4th Year	Final Year	Interns	Total (%)
Have you heard of the term *Periodontitis*?	Yes	47 (88.7%)	54 (98.2%)	23 (100%)	85 (97.7%)	209 (95.9%)
	No	6 (11.3%)	1 (1.8%)	0 (0.0%)	2 (2.3%)	9 (4.1%)
Total						218

### Common signs and symptoms

When asked about the symptoms caused by periodontitis, the majority of them answered Bad Breath (182; 83.5%), Gum Recession (171; 78.4%), Bleeding Gums (186; 85.3%), and Loose Tooth (155; 71.1%). More than 80% of every batch marked Gum Recession as a symptom of Periodontitis, except for 3rd year students; in which only 28 (52.8%) students marked it as one of the right options. Similarly, more than the majority from every batch marked loose Tooth as one of the correct options, except for 3rd year students; in which only 25 (47.2%) students marked it as a symptom of periodontitis [Table 3].

**Table 3:** Common signs and symptoms (N=218).

Sign/Symptom	3rd Year	4th Year	Final Year	Interns	Total (%)
**Bad Breath**	38 (71.6%)	43 (78.2%)	21 (91.3%)	80 (91.2%)	182 (83.5%)
**Oral Candidiasis**	11 (20.7%)	21 (38.2%)	6 (26.1%)	18 (20.7%)	56 (25.7%)
**Gum Recession**	28 (52.8%)	44 (80.0%)	23 (100%)	76 (87.4%)	171 (78.4%)
**Tooth Fracture**	11 (20.7%)	16 (29.1%)	4 (17.4%)	16 (18.4%)	47 (21.6%)
**Tooth Decay**	24 (45.3%)	26 (47.3%)	13 (56.5%)	43 (49.4%)	106 (48.6%)
**Bleeding Gums**	35 (66.0%)	47 (85.5%)	22 (95.7%)	82 (94.3%)	186 (85.3%)
**Tooth Loss**	18 (34.0%)	26 (47.3%)	12 (52.2%)	44 (50.6%)	100 (45.9%)
**Loose Tooth**	25 (47.2%)	39 (70.9%)	19 (82.6%)	72 (82.8%)	155 (71.1%)

### Risk factors

When asked about the risk factors for periodontitis, more than two-thirds of the participants reported socioeconomic status (153; 70.2%), smoking (189; 86.7%), and tooth decay (153; 70.2%). More than two-thirds of students from every batch selected socioeconomic status, except for 3rd year students; in which only 27 (50.9%) students identified it as a risk factor for periodontitis. Similarly, more than half of the students from every batch selected Ehlers-Danlos syndrome, except for 3rd year students; in which only 19 (35.8%) students marked it as a risk factor for periodontitis. Additionally, more than one-third of every batch selected Pregnancy, except for 3rd and 4th year students; in which only 9 (17%) and 12 (21.8%) students respectively marked it as a risk factor for periodontitis [Table 4].

**Table 4:** Risk factors (N=218).

Risk Factor	3rd Year	4th Year	Final Year	Interns	Total (%)
**Oral Candidiasis**	38 (71.7%)	36 (65.5%)	15 (65.2%)	43 (49.4%)	132 (60.6%)
**Socioeconomic Status**	27 (50.9%)	44 (80.0%)	17 (73.9%)	65 (74.7%)	153 (70.2%)
**Ehlers Danlos Syndrome**	19 (35.8%)	28 (50.9%)	12 (52.2%)	47 (54.0%)	106 (48.6%)
**Papillon LeFevre Syndrome**	11 (20.8%)	13 (23.6%)	3 (13.0%)	22 (25.3%)	49 (22.5%)
**Smoking**	40 (75.5%)	49 (89.1%)	19 (82.6%)	81 (93.1%)	189 (86.7%)
**Sedentary Lifestyle**	22 (41.5%)	20 (36.4%)	8 (34.8%)	38 (43.7%)	88 (40.4%)
**Pregnancy**	9 (17.0%)	12 (21.8%)	10 (43.5%)	31 (35.6%)	62 (28.4%)
**Tooth Decay**	33 (62.3%)	38 (69.1%)	16 (69.6%)	66 (75.9%)	153 (70.2%)

### Systemic conditions linked with periodontitis

When asked about systemic conditions/diseases linked with periodontitis, more than two-thirds of the participants answered Alzheimer’s disease (104; 66.1%) and diabetes mellitus (167; 76.6%). Also, more than one-third of the participants answered Systemic Lupus Erythematosus (94; 43.1%). The majority of the students from every batch selected Alzheimer’s disease, except for 3rd and 4th-year students; in which 16 (30.2%) and 24 (43.6%) students respectively marked it to have a link with periodontitis. Similarly, more than three-fourths of the students from every batch selected Diabetes Mellitus, except for 3rd year students; in which only 30 (56.6%) students marked it as having a link with Periodontitis. Additionally, compared with the other groups, 25 (47.2%) students from the 3rd year selected osteoarthritis to be linked with periodontitis [Table 5].

**Table 5:** Systemic conditions linked with periodontitis (N=218).

Condition/Disease	3rd Year	4th Year	Final Year	Interns	Total (%)
**Alzheimer’s Disease**	16 (30.2%)	24 (43.6%)	14 (60.9%)	50 (57.5%)	104 (66.1%)
**Rheumatoid Arthritis**	17 (32.1%)	21 (38.2%)	9 (39.1%)	27 (31.0%)	74 (33.9%)
**Systemic Lupus Erythematosus**	19 (35.8%)	24 (43.6%)	14 (60.9%)	37 (42.5%)	94 (43.1%)
**Sarcoidosis**	17 (32.1%)	17 (30.9%)	9 (39.1%)	27 (31.0%)	70 (32.1%)
**Adverse Pregnancy Outcome**	11 (20.8%)	12 (21.8%)	7 (30.4%)	26 (29.9%)	56 (25.7%)
**Osteoarthritis**	25 (47.2%)	20 (36.4%)	6 (26.1%)	16 (18.4%)	67 (30.7%)
**Ectodermal Dysplasia**	14 (26.4%)	23 (41.8%)	8 (34.8%)	30 (34.5%)	75 (34.4%)
**Chronic Kidney Disease**	6 (11.3%)	16 (29.1%)	6 (26.1%)	34 (39.1%)	62 (28.4%)
**Diabetes Mellitus**	30 (56.6%)	42 (76.4%)	19 (82.6%)	76 (87.4%)	167 (76.6%)
**Cardiovascular Disease**	8 (15.1%)	17 (30.9%)	12 (52.2%)	33 (37.9%)	70 (32.1%)
**Hospital-Acquired Pneumonia**	10 (18.9%)	8 (14.6%)	5 (21.7%)	26 (29.9%)	49 (22.5%)

### Referral to dentists

when asked if they would routinely refer patients with Type II Diabetes Mellitus to the dentist, one-third of the participants (149; 68.3%) said ‘yes’. The highest number of students who disagreed with the routine referral of a patient with Type II Diabetes Mellitus to a dentist were from the 3rd year (21; 39.6%) [Table 6].

**Table 6:** Referral to dentist (N=218).

Response	3rd Year	4th Year	Final Year	Interns	Total (%)
**Yes**	32 (60.4%)	38 (69.1%)	18 (78.3%)	61 (70.1%)	149 (68.3%)
**No**	21 (39.6%)	17 (30.9%)	5 (21.7%)	26 (29.9%)	69 (31.7%)

### Knowledge of periodontitis

When asked about how they would rate their knowledge of periodontitis, the highest number of responses, 64 (29.4%) students, marked it as ‘Poor’. However, 2 (0.9%) students indicated that their knowledge of Periodontitis was ‘Excellent’. More than one-third of the students from 3rd and 4th-year students, 24 (45.3%) and 23 (41.8%) students respectively marked their knowledge of Periodontitis as ‘poor’. Also, more than half of the students from the final year, significantly more than other batches, 12 (52.2%) students answered to have a ‘good’ knowledge of periodontitis [Table 7].

**Table 7:** Knowledge of periodontitis (N=218).

Knowledge Rating	3rd Year	4th Year	Final Year	Interns	Total (%)
**Poor**	24 (45.3%)	23 (41.8%)	2 (8.7%)	15 (17.2%)	64 (29.4%)
**Fair**	15 (28.3%)	14 (25.5%)	4 (17.4%)	19 (21.8%)	52 (23.9%)
**Neutral**	10 (18.9%)	14 (25.5%)	5 (21.7%)	33 (37.9%)	62 (28.4%)
**Good**	3 (5.7%)	4 (7.3%)	12 (52.2%)	19 (21.8%)	38 (17.4%)
**Excellent**	1 (1.9%)	0 (0.0%)	0 (0.0%)	1 (1.1%)	2 (0.9%)

### Awareness of dental conditions

When asked whether having an awareness of commonly occurring dental conditions and diseases would help their practice of medicine, nearly all of the participants, 211 (96.8%) students, said ‘yes’. The highest number of students, 4 (7.3%) students, saying ‘no’ to the above question were from the 4th year [Table 8].

**Table 8:** Awareness of dental conditions (N=218).

Response	3rd Year	4th Year	Final Year	Interns	Total (%)
Yes	52 (98.1%)	51 (92.7%)	23 (100%)	85 (97.7%)	211 (96.8%)
No	1 (1.9%)	4 (7.3%)	0 (0.0%)	2 (2.3%)	7 (3.2%)

### Preferred learning method

When asked about their preferred format to learn more information on periodontitis, video (147; 67.4%) followed by information pack (86; 39.4%), lectures (81; 37.2%), and website (62; 28.4%) were the preferred choices [Table 9].

**Table 9:** Preferred learning method (N=218).

Preferred Format	3rd Year	4th Year	Final Year	Interns	Total (%)
**Lectures**	22 (41.5%)	21 (38.2%)	7 (30.4%)	31 (35.6%)	81 (37.2%)
**Information Pack**	13 (24.5%)	22 (40.0%)	7 (30.4%)	44 (50.6%)	86 (39.4%)
**Video**	39 (73.6%)	33 (60.0%)	14 (60.9%)	61 (70.1%)	147 (67.4%)
**Website**	16 (30.2%)	14 (25.5%)	4 (17.4%)	28 (32.2%)	62 (28.4%)

## Discussion

This study highlights significant gaps in the awareness and knowledge of periodontal disease and its systemic implications among medical students in Nepal. Compared with dental education, the MBBS curriculum as outlined by Nepal Medical Council in Nepal does not appear to place significant emphasis on periodontal health [13]. There is no specific mention of periodontal health or dentistry in these standards. In contrast, dental education likely dedicates more curricular time to oral health topics, including periodontal diseases and their management, given the specialized nature of dentistry compared with the broad scope of medical education. However, the MBBS program in Nepal, while comprehensive, does not seem to prioritize periodontal health to the same degree as undergraduate dental curricula [13,14]. Thus, this study is important in this field, as it is the first of its kind to explore the understanding and awareness of periodontitis and its associated factors among medical students in Nepal.

The majority of respondents (95.9%) were aware of periodontitis, which represents a higher level of awareness compared to a previous study conducted on this topic [6,15]. Although common signs like bleeding gums (85.3%) and gum recession (78.4%) were widely recognized, many respondents incorrectly identified tooth decay and oral candidiasis as symptoms of periodontitis. This reflects confusion about the pathophysiology of periodontitis compared to other oral diseases, such as dental caries. Misunderstandings about fundamental concepts like these could limit medical professionals’ ability to identify oral health issues and provide appropriate referrals.

While respondents demonstrated an awareness that smoking and diabetes mellitus are risk factors for periodontitis, as supported by previous studies, this does not necessarily indicate a full understanding of their specific roles in the disease’s development [16–18]. This is because smoking and diabetes are also well-established risk factors for many other chronic non-communicable diseases. Furthermore, many participants incorrectly identified tooth decay, oral candidiasis, and Ehlers-Danlos syndrome as risk factors for periodontitis. These misconceptions highlight a lack of in-depth knowledge about the specific etiological factors underlying this condition, emphasizing the need for more comprehensive education on this topic.

The survey assessed students’ awareness of the relationship between periodontitis and systemic diseases, including conditions with well-established associations and those without strong evidence of a link. While the majority of respondents correctly identified diabetes mellitus (76.6%) as being associated with periodontitis, only 32.1% recognized its connection with cardiovascular diseases—despite these being two of the most strongly supported associations in the literature [15,19] Additionally, more than half of the participants incorrectly associated periodontitis with Alzheimer’s disease (66.1%) and nearly half with systemic lupus erythematosus (43.1%), conditions without robust evidence of a causal relationship. This indicates that students had a poorer understanding of associations where the evidence is less consistent, highlighting the need for more targeted education on oral-systemic health connections as felt in previous studies in UK and Saudi Arabia [12,20]. These knowledge gaps suggest a need for targeted interventions in medical education to clarify the distinctions and intersections between oral and systemic health.

Although medical students do not require the same depth of education on oral diseases as dental students, incorporating basic instruction on these topics is essential. This should cover the signs and symptoms of common oral diseases, their systemic health impacts, and the interplay between systemic and oral health in patient management. Introducing this content during undergraduate training is critical, as clinicians’ knowledge tends to decline post-graduation, underscoring the importance of building this foundation early.

Incorporating oral health topics into medical education has been shown to improve knowledge and patient care outcomes. For example, NHS England recommends referring diabetic patients to dental services for periodontal screening, a practice that has improved glycemic control and reduced complications [21]. However, no formal referral protocol exists in Nepal for connecting diabetic patients with dental care. Although the majority of medical students surveyed (68.3%) indicated they would refer diabetic patients to a dentist, the absence of a structured referral pathway represents a missed opportunity to ensure essential dental care for this high-risk group. A similar gap was highlighted in a survey conducted among Nepalese dentists, emphasizing the need for coordinated interprofessional strategies [22].

The majority of students (96.8%) believed that understanding common dental conditions would improve their medical practice. A study at another Nepalese university similarly found that undergraduate medical students lacked fundamental knowledge about oral health practices [23]. This highlights the vital connection between systemic and oral health, where changes in one can influence the other. Educating students on this relationship is essential to fostering a comprehensive, patient-centered approach and shaping future doctors’ attitudes toward the importance of oral health in overall healthcare.

Delivering such education within an already congested curriculum requires innovative teaching strategies. Students in this study expressed a preference for multimedia formats, such as videos (67.4%) and information packs (39.4%), which can be used to create flexible e-learning resources. These tools can incorporate pre-recorded lectures, interactive modules, and updated evidence-based guidelines, allowing students to learn at their own pace while addressing knowledge gaps effectively. Similar approaches have been successfully implemented in other contexts and may be adapted to the Nepalese setting [24].

Notably, student-led initiatives have recently been launched to improve awareness of this topic among medical and dental students. Education should not be limited to academia, as general dental practitioners can also play a crucial role in communicating the importance of oral health to their medical counterparts. Opportunities exist for involvement with local diabetes clinical networks. Another approach to consider is providing brief information or signposting educational resources in correspondence with medical doctors. This can help raise awareness and encourage a more holistic, patient-centered approach to managing systemic and oral health.

The role of medical doctors is not to diagnose and treat periodontitis, or indeed, most other dental conditions. However, a basic level of understanding concerning them is still required by medical doctors for effective, interprofessional communication to highlight the importance of oral health and ultimately to provide holistic care for patients.

The results of this study are comparable to those of most similar studies carried out in other countries. These studies tended to conclude that the knowledge of medical practitioners and students on periodontitis is limited. One similar study from Saudi Arabia reported adequate awareness; however, the responses from medical students could not be isolated from those of other student groups in that study. Nonetheless, all the studies agreed that further education on periodontitis would be beneficial for medical practitioners and students [25–27].

This study revealed that the 3rd year students have less knowledge regarding the relationship between systemic diseases and periodontal health than the 4th year, final-year, and intern students. There seems to be a need to make medical students aware of the relationship between systemic and periodontal health from the beginning of their clinical years as they start interacting with patients directly. With background knowledge of the severity of oral manifestations of systemic diseases, medical students as well as physicians can refer patients for early dental consultation which will eventually reduce the financial burden on patients and provide a better prognosis for oral health.

This study has several strengths and limitations. It provides the first evidence of medical students’ awareness of periodontal disease in Nepal, offering a foundation for future research and curriculum development. However, its single-center design limits the generalizability of findings. The use of closed-ended questions may have introduced response bias, with participants potentially guessing or providing socially desirable answers [28]. Expanding the study to multiple institutions and including qualitative methods, such as focus groups or interviews, could provide deeper insights and a broader perspective.

## Conclusion

Despite the study’s limitations, the findings suggest that medical students’ awareness of periodontitis and its relationship with systemic health is limited. The researchers recommend increased educational focus on periodontitis and oral diseases in general. They also suggest the Nepal Medical Council to incorporate more specific oral health learning outcomes in medical curricula.

Researchers do not advocate that medical doctors take over dentists’ roles but rather emphasize the need for sufficient awareness to allow appropriate patient management through brief interventions, signposting, or referrals. Ultimately, both medical doctors and dentists strive for a holistic, patient-centered approach, which requires appropriate training and education on various diseases and their systemic impacts, ideally starting from the undergraduate level.

## Suppporting information

S1. File Data. (XLSX)
